# Sex differences in the association between latent class of lifestyle and disability among older adults in China

**DOI:** 10.1186/s12877-021-02087-z

**Published:** 2021-03-18

**Authors:** Zaixing Shi, Jianlin Lin, Jian Xiao, Ya Fang

**Affiliations:** 1 State Key Laboratory of Molecular Vaccinology and Molecular Diagnostics, School of Public Health, Xiamen University, Xiamen, China; 2 Key Laboratory of Health Technology Assessment of Fujian Province, School of Public Health, Xiamen University, Xiamen, China

**Keywords:** Aging, Disability, Lifestyle, Sex differences, Latent class analysis

## Abstract

**Background:**

A healthy lifestyle may prevent disability for older adults. But research to date is limited to a single lifestyle behavior and ignore sex difference in the lifestyle-disability association. This study aimed at identifying sex-specific latent classes of lifestyle and their relationship with disability among older Chinese adults.

**Methods:**

Data were obtained from adults aged 65 years or above in the 2018 Chinese Longitudinal Healthy Longevity Survey, a nationally representative sample of older adults in China. We used latent class analysis to categorize participants into subgroups based on three dimensions of lifestyle factors: health behaviors, psychological wellbeing, and social engagement. Disability was assessed by the activities of daily living (ADL). Multivariable logistic regression was used to evaluate the associations between the latent lifestyle classes and disability.

**Results:**

A total of 15,771 older adults were included in this analysis, of whom 56% were women and 66% aged 80 years or above. We identified four latent lifestyle classes among older women: “Health Promoting” (28%), “Isolated and Health Harming” (34%), “Restless and Dismal” (21%), and “Restless” (17%). A different set of four lifestyle classes were identified in older men: “Health Promoting” (21%), “Isolated and Health Harming” (26%), “Restless and Dismal” (20%), and “Discordant” (33%). Compared with the “Health Promoting” class, the “Isolated and Health Harming” class (OR = 1.88, 95% CI: 1.46–2.43) and the “Restless and Dismal” class (OR = 1.67, 95% CI: 1.27–2.20) had higher risk of disability in women. The “Discordant” class had lower risk of disability in men (OR = 0.52, 95% CI: 0.37–0.72).

**Conclusions:**

Our analyses revealed different lifestyle patterns for older women and men in China. Sex differences in the associations between lifestyle and disability need to be considered when formulating interventions to prevent disability.

**Supplementary Information:**

The online version contains supplementary material available at 10.1186/s12877-021-02087-z.

## Background

Disability refers to temporary or permanent loss of physical or mental function, according to the World Health Organization (WHO) [1]. Disability is a major challenge for healthy aging in China. It is estimated that the disability rate of the middle-aged and older adults in China was 21.7% in 2014, higher than the prevalence in the United States (16.5%), Austria (13.7%) and other western countries [2]. By 2050, there would be 91.4 million disabled adults aged over 65 years in China, with a disability rate of 26.44% [3]. Previous studies among older Chinese adults suggest that disability is associated with socioeconomic status, living arrangement, self-rated health, and lifestyle [4–6]. Recently, various lifestyle factors have been associated with preventing functional decline for older adults [7]. For example, non-smoking, moderate but not heavy drinking, physical activity, and a healthy weight are important promoters of functional independence in older adults [8]. Psychological and social factors are also associated with disability. For example, depression is a risk factor for declining psychological functions in older adults [9] and that less social support and social engagement are risk factors for worsening social functions [10]. Collectively, these evidence suggest that lifestyle as a whole may affect daily functioning.

However, previous studies are generally limited to a single health behavior or a few behaviors that are traditionally considered as “lifestyle factors,” such as smoking, alcohol drinking, and exercise, while the psychological and social aspects of lifestyle are often ignored. A single lifestyle behavior may have little impact on disability and is not informative for developing targeted lifestyle intervention. For example, people who smoke and drink alcohol are at increased risk of disability, but there may be groups who do not smoke and drink but have poor psychological status, who are also at increased risk of disability. Therefore, it is important to identify the combination patterns of different lifestyles to develop comprehensive strategies to improve lifestyle and prevent disability. The WHO’s International Classification of Functioning, Disability, and Health (ICF) [1] suggests that a broad range of personal factors, such as exercise and alcohol drinking, and social factors, such as social engagement, may affect an individual’s physical function. The ICF provides a framework for understanding lifestyle patterns associated with disability.

Several studies have started to understand the distinct patterns of lifestyle in older adults. For example, a previous study categorized retirees into a group with a healthy lifestyle and a group with a less healthy lifestyle according to their diet, alcohol consumption, cigarette smoking, physical activity, and TV viewing behaviors, and explored the relationship between lifestyle patterns and sociodemographic characteristics [11]. Another Brazilian study divided older adults into three latent classes: “Healthy”, “Poor diet and physical activity”, “Smoking and binge drinking”, according to their fruits and vegetable consumption, alcohol drinking, cigarette smoking, physical activity, and TV viewing behaviors [12].

However, few studies have considered sex differences when exploring latent classes of lifestyle, although previous studies have found differences in lifestyle between men and women [13]. Moreover, few studies have evaluated the relationship between different lifestyle patterns and the risk of disability among older adults. To fill in this gap, this analysis aimed to 1) identify the latent classes of lifestyle among older women and men in China based on a nationally representative sample, and 2) analyze the associations between the latent lifestyle classes and disability by sex. The findings will facilitate the development of interventions to promote active and healthy aging in older adults.

## Methods

### Data source

We used the 2018 wave of the Chinese Longitudinal Healthy Longevity Survey (CLHLS), with 15,874 participants. The CLHLS is the first study to investigate factors that impact the health of older adults in China from a multidimensional perspective, which has national representation. Detailed study design of the CLHLS has been reported in a previous report [14]. A total of 15,771 participants aged ≥65 years were included in this study, after excluding those with missing lifestyle indicators.

### Measures

We derived latent lifestyle classes based on 16 lifestyle factors reflecting health behaviors, psychological wellbeing, and social engagement (Supplemental Table 1, Additional File 1). These lifestyle factors were considered predictors of disability according to ICF [1]. All data were collected through face-to-face interview by trained personnel with medical background using a standard questionnaire.

#### Health behaviors

Measures of health behaviors included the following items: sleep quality, sleep duration, consumption of vegetables, consumption of fruits, alcohol drinking, smoking, exercise, physical examination, frequency of tooth brushing per day, and chronic disease management. Specifically, sleep quality was self-reported, categorized as good, moderate, and bad. Sleep duration records the usual number of hours of sleep and was categorized as less than 7 h, 7 to 8 h, and more than 8 h. Consumption of fruits and vegetables were categorized as sufficient (quite often, every day, or almost every day) and insufficient (occasionally, rarely, or never). Alcohol drinking, smoking, exercise, and regular physical examination, were categorized as binary variables (yes or no). The frequency of tooth brushing measures how often older adults brush their teeth and was categorized as less than twice per day and twice or more per day. Chronic disease management was evaluated by the diagnosis and treatment status of three common chronic diseases, including hypertension, diabetes, and dyslipidemia (not diagnosed, diagnosed but not taking medicine, and diagnosed and taking medicine).

#### Psychological wellbeing

The indicators of psychological wellbeing were evaluated by the Chinese version of the Center for Epidemiologic Studies Depression Scale (CES-D) [15] and the Generalized Anxiety Disorder Questionnaire (GAD-7) Scale [16]. The CES-D has 9 items, including “Are you bothered by things that don’t usually bother you?” , “Do you have trouble focusing on what you were doing?”, “Do you feel sad, blue, or depressed?”, “Do you feel the older you get, the more useless you are, and have trouble doing anything?”, “Do you feel hopeful about the future?”, “Do you often feel fearful or anxious?”, “Are you as happy as when you were younger?”, “Do you often feel lonely and isolated?”, and “Do you feel you could not get going?” Each item was categorized as never, seldom, sometimes, often, and always. The CES-D scale showed good internal consistency in the current sample, with a standardized Cronbach’s α of 0.82. A score ranging from 1 to 5 was assigned to each response, adding up to a total score of 45, with a higher score suggesting more depressive symptoms. Depression was categorized as no (score < 27) and yes (score ≥ 27), according to the 60th percentile.

The GAD-7 scale has 7 items, including “Feeling uneasy, worried and annoyed”, “Can’t stop or can’t control worry”, “Is worried too much about all kinds of things”, “Is very nervous and it is difficult to relax”, “Is very anxious, so you can’t sit still”, “Becomes easy to get annoyed or easily irritated”, and “Feels like something terrible happens”. Each item was categorized as never, for several days, more than half of days, and almost every day. A score ranging from 1 to 4 was assigned to each response, adding up to a total of 28. Anxiety was categorized as no (score < 14) and yes (score ≥ 14), according to the 50th percentile. The GAD-7 scale demonstrated excellent internal consistency in this sample, with a standardized Cronbach’s α of 0.92.

#### Social engagement

Social engagement was evaluated by structural and functional relationships [17]. The structural relationships were measured by five items: in marriage (1 = “yes”, 0 = “no”), having children (1 = “yes”, 0 = “no or otherwise”), playing cards and/or mah-jongg (1 = “≥ once per month”, 0 = “otherwise”), attending social activities (1 = “≥ once per month”, 0 = “otherwise”), and visiting and interacting with friends (1 = “≥ once per month”, 0 = “otherwise”). The total score of structured social interaction is a sum of 5, and the lack of structural relationships was defined as a score ≤ 2. The functional relationships were evaluated by 3 items: “To whom do you usually talk most frequently in daily life?”, “To whom do you talk first when you need to tell something of your thoughts?” and “Who do you ask first for help when you have problems/difficulties?” For the first item, participants could select up to three persons from ten types of relationships (kinship and friends/neighbors), but for the second and third items, participants could select up to two persons. For each item, the first, second, and third selections were assigned a score of 3 to 1, respectively. The total score of the three items ranges from 0 to 16, which was categorized as lacking a functional relationship (score ≤ 10) or not (score > 10).

#### Covariates

Sociodemographic characteristics were included as covariates, including age (65–79, 80+), marital status (in marriage, not in marriage), residence (urban, rural), living status (alone, with others), education (illiterate, literate or primary school, and junior high and above), occupation before age 60 (agriculture, professional/managerial, and others), household income (lower than 6000 Yuan, 6000–19,999 Yuan, 20,000–39,999 Yuan and higher than 40,000 Yuan), and self-rated health (very bad, bad, average, good, and very good). Cognitive function was evaluated by the Chinese version of the mini-mental state examination (MMSE; 0–30 scores) [18, 19], which showed excellent internal consistency in this sample (standardized Cronbach’s α = 0.87). Cognitive impairment was defined as an MMSE score ≤ 19 for illiterate participants, MMSE score ≤ 22 for those who were literate or only attended primary school, and MMSE score ≤ 26 for those with a junior high school education or above [20].

#### Disability

Disability was assessed by the Chinese version of the Katz’s activities of daily living (ADL) scale [21, 22], which showed excellent internal consistency in this sample (standardized Cronbach’s α = 0.91). The scale assessed one’s capability of bathing, dressing, toilet, indoor transfer, continence, and eating. Participants were asked if they need assistance in performing the 8 activities. Disability was defined as requiring assistance in any of the ADLs [23, 24].

### Statistical analyses

First, latent class analysis (LCA) was used to identify the latent classes according to lifestyle factors among older adults. The LCA estimates the distribution of latent lifestyle classes and conditional probabilities of lifestyle variables for each latent class [25]. We assumed that data were missing at random in this sample. The expectation-maximization algorithm makes it possible to estimate latent classes when some lifestyle variables had missing values [26]. The latent classes of lifestyle of older adults were named according to the distinctive conditional probability of lifestyle variables in each latent class. LCA models with 2 to 10 classes were performed to find the best-fitting model. Model selection was based on the Bayesian information criterion (BIC), adjusted BIC (aBIC), Akaike’s information criterion (AIC), and consistent Akaike’s information criterion (cAIC), with a lower value indicating better fit [27]. In addition to the model-fitting metrics, the simplicity and interpretability of the model were also considered.

Second, we tested measurement invariance of LCA across sex. Measurement invariance assumes that the latent classes are comparable across different subpopulations. In the test of measurement invariance, models with equal and unequal parameters were fitted respectively and compared using the *χ*^2^ test [25]. If the assumption of measurement invariance is valid, the distribution of latent classes and conditional probabilities will be equal between sex. Otherwise, LCA should be performed for men and women separately.

Finally, multivariable logistic regression model was used to examine the associations of latent lifestyle classes with disability. The multivariable logistic regressions controlled for potential confounders that have been associated with both lifestyle and disability [11, 28], including age, marital status, residence, living status, education, main occupation before 60, household income, and self-rated health (Molde 1). As the study is focusing on older adults, it is possible that a proportion of respondents had some form of cognitive impairment such as dementia. Cognitive impairment may confound the association between lifestyle and disability as it could be associated with a) a person’s everyday functioning or disability, b) lifestyle factors such as smoking and drinking [29–31], and c) the reliability of survey results. Therefore, we additionally adjusted for cognitive impairment status to assess its impact on the observed association between lifestyle classes and disability (Model 2). Due to the low response rate to the MMSE scale (59.7% responded), we conducted multiple imputation for missing MMSE score to avoid potential selection bias if participants with missing cognitive assessment data were excluded from analyses. Following Rubin’s approach [32], we generated 50 imputed datasets and fitted the Model 2 on each imputed dataset, which were then averaged to estimate the summary coefficients and standard errors of Model 2. Furthermore, because the literacy rate is low among the oldest old in China [33], the next generation may have higher education. To evaluate the robustness of our results across populations with varying literacy rates, we performed sensitivity analyses to evaluate the association between lifestyle and disability according to age groups (65–74, 75–84, and 85+ years). Data collection and management were conducted in R version 4.0.2. LCA was performed in SAS (version 9.4) [34]. Multiple imputation was performed using R package “mice” [35, 36]. *P* < 0.05 was regarded as statistically significant.

## Results

### Sample characteristics

Of the 15,771 participants, 6869 (44%) were men and 8902 (56%) were women. The majority of participants aged 80 years or above (66%), illiterate or only attended primary education (81%), and reported an annual household income of less than 40,000 Chinese Yuan, or approximately 5800 US dollars (69%) (Supplemental Table 2, Additional File 1). More than 28% of older adults met the criteria for disability.

### Identification of latent classes of lifestyle

Among models with 2 to 10 classes, a four-class model was selected (Supplemental Table 3, Additional File 1) for both women and men. The results of measurement invariance show that the conditional probability of lifestyle indicators between men and women was not equal (Supplemental Table 4, Additional File 1). Therefore, the LCA was implemented for men and women separately.

**Fig. 1:**
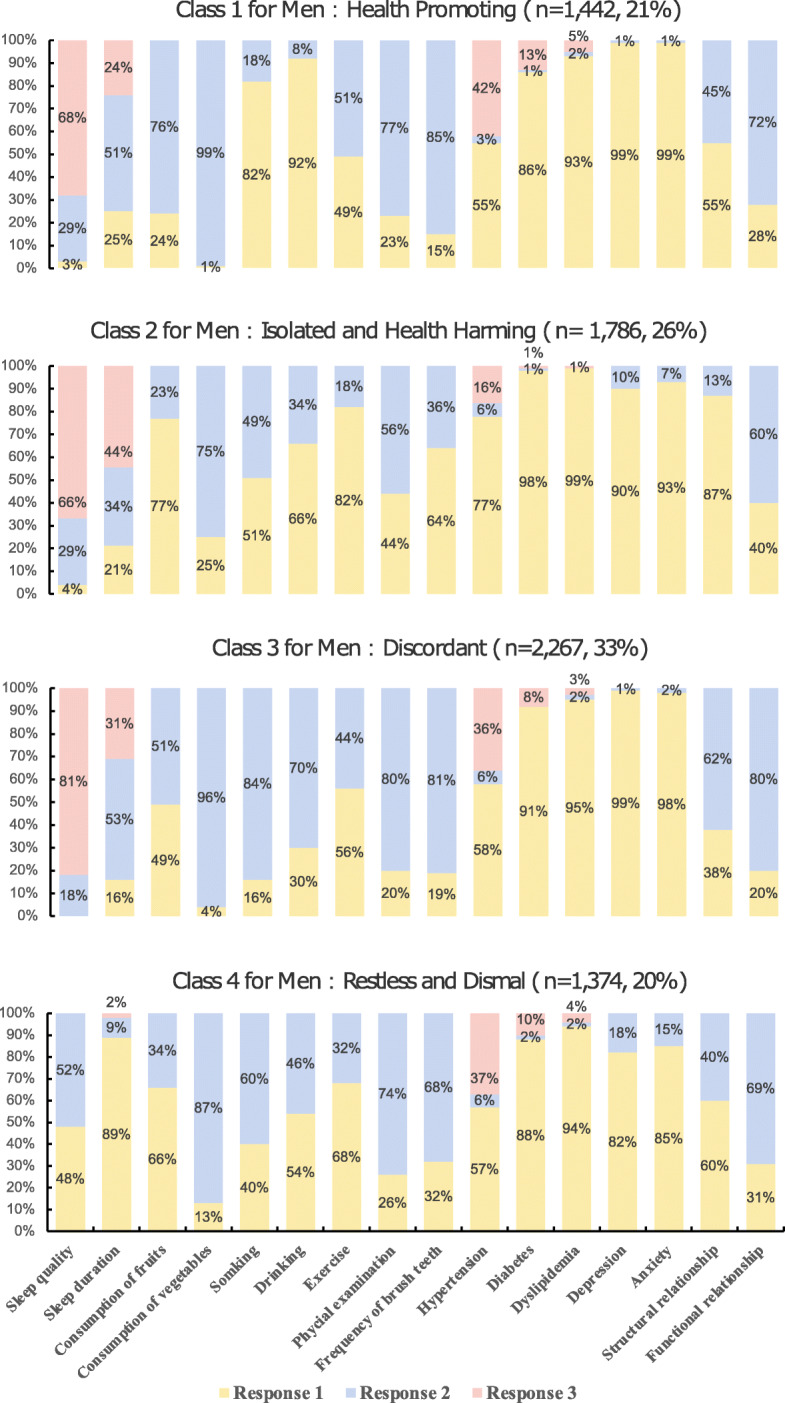
The distribution of latent classes and conditional probability for men. For sleep quality: response 1 = bad, response 2 = moderate, response 3 = good. For sleep time: response 1 = less than 7 h, response 2 = 7 to 8 h, response 3 = more than 8 h. For consumption of fruits and vegetable: response 1 = insufficient, response 2 = sufficient. For hypertension, diabetes, and dyslipidemia, response 1 = not diagnosed, response 2 = diagnosed but not taking medicine, response 3 = diagnosed and taking medicine. For other binary variables, response 1 = no and response 2 = yes

**Fig. 2:**
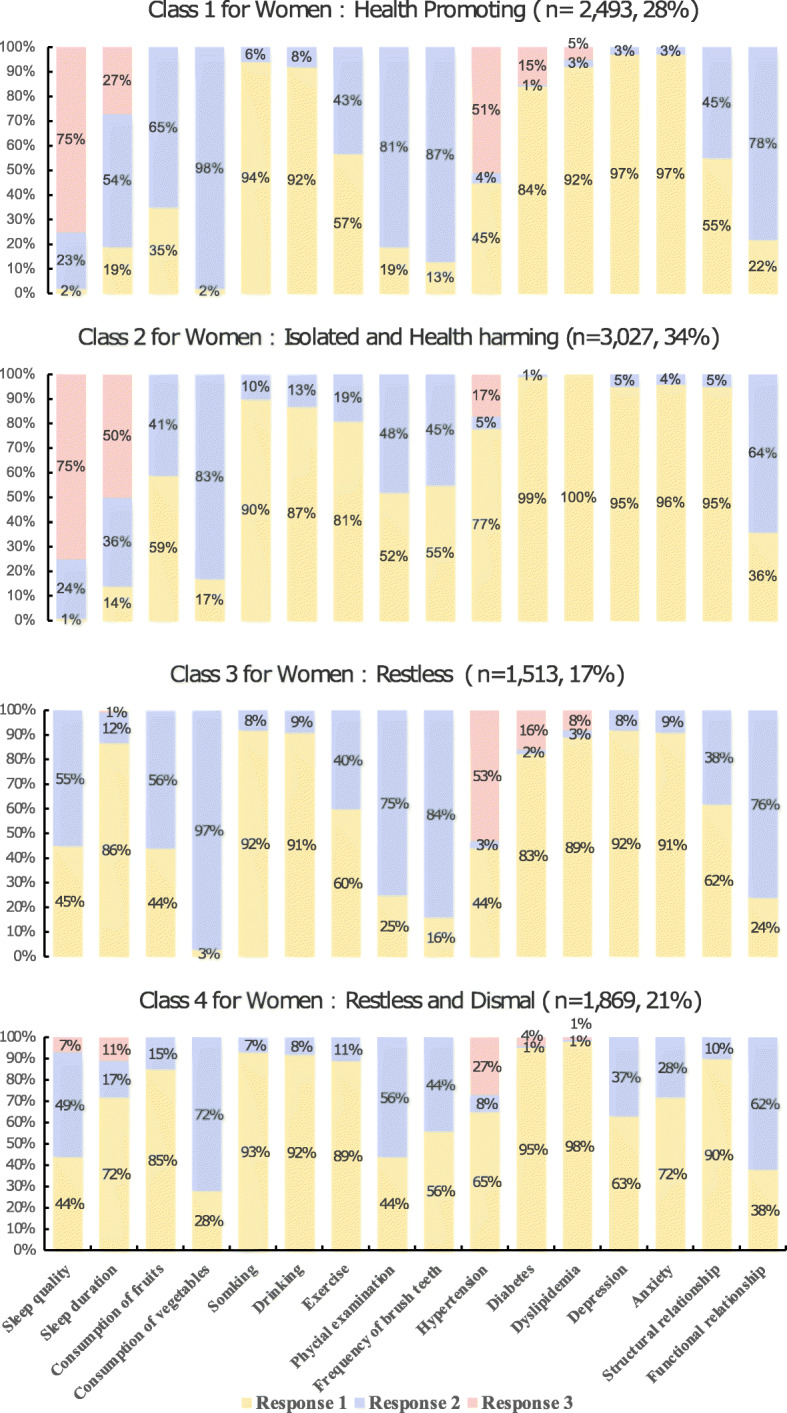
The distribution of latent classes and conditional probability for women. For sleep quality: response 1 = bad, response 2 = moderate, response 3 = good. For sleep time: response 1 = less than 7 h, response 2 = 7 to 8 h, response 3 = more than 8 h. For consumption of fruits and vegetable: response 1 = insufficient, response 2 = sufficient. For hypertension, diabetes, and dyslipidemia, response 1 = not diagnosed, response 2 = diagnosed but not taking medicine, response 3 = diagnosed and taking medicine. For other binary variables, response 1 = no and response 2 = yes

The latent lifestyle classes were named according to the most distinctive conditional probability of lifestyle variables in each class (Fig. 1 and Fig. 2). For women, the first class (*n* = 2493, 28%) tended to report good sleep quality, exercise, regular physical examination, good structural relationship and function relationship. Thus, the first class was labeled as “Health Promoting”. The second class (*n* = 3027, 34%) was more likely to report smoking and alcohol drinking, and lacked structural relationships. Thus, the second class was labeled as “Isolated and Health Harming”. The third class (*n* = 1513, 17%) had poorer sleep quality and shorter sleep time, thus was labeled as “Restless”. The last class (*n* = 1869, 21%) was similar to the “Restless” class but also reported higher depression and anxiety. Thus, to distinguish from those with poor sleep only, the fourth class was labeled as “Restless and Dismal.”

For men, the first class (*n* = 1442, 21%) was more likely to exercise, brushing teeth, receiving physical examination, and had good function relationship. Thus, the first class was labeled as “Health Promoting”. The second class (*n* = 1786, 26%) was more likely to report insufficient consumption of fruits and vegetables, and lacked structural relationships. Thus, the second class was labeled as “Isolated and Health Harming.” The third class (*n* = 2267, 33%) tended to have higher probabilities of smoking, alcohol drinking, and no exercise, but was less likely to have depression or anxiety, and reported better structural relationship and functional relationship. Thus, the third class was labeled as “Discordant.” The fourth class (*n* = 1374, 20%) was characterized by poor sleep quality, short sleep duration, depression and anxiety. Thus, the fourth class was labeled as “Restless and Dismal”.

### Characteristics of latent lifestyle classes

The sociodemographic characteristics according to latent lifestyle classes are shown in Table 1 and Table 2. There were significant differences in age, marital status, education, main occupation before 60, household income, self-rated health across latent lifestyle classes. Participants with and without disability also showed significant differences in these sociodemographic characteristics (Supplemental Tables 5, Additional File 1).

**Table 1:** Sociodemographic characteristics of latent lifestyle classes for men (*N* = 6869)

Variables	Total (***N*** = 6869)	Health Promoting (***N*** = 1442)	Isolated and Health Harming (***N*** = 1786)	Discordant (***N*** = 2267)	Restless and Dismal (***N*** = 1374)	***P***-value
	N(%)	N(%)	N(%)	N(%)	N(%)	
**Age**						< 0.001
65–79	2698 (39)	352 (20)	1264 (55)	495 (38)	587 (40)	
≥ 80	4171 (61)	1431 (80)	1050 (45)	804 (62)	886 (60)	
**Residence area**						< 0.001
Rural area	2978 (43)	865 (49)	997 (43)	446 (34)	670 (45)	
Urban area	3891 (57)	918 (51)	1317 (57)	853 (66)	803 (55)	
**Current marital status**						< 0.001
Not in marriage	2813 (39)	1154 (20)	596 (55)	491 (38)	572 (40)	
In marriage	3948 (61)	582 (80)	1693 (45)	798 (62)	875 (60)	
**Living status**						< 0.001
Alone	227 (4)	65 (5)	40 (2)	72 (6)	50 (4)	
With others	5585 (96)	1346 (95)	2009 (98)	1068 (94)	1162 (96)	
**Education**						< 0.001
Illiterate	1574 (27)	647 (44)	356 (18)	216 (20)	355 (29)	
Literate or primary school	2504 (43)	631 (43)	905 (46)	432 (39)	536 (44)	
Junior high or above	1681 (29)	203 (14)	689 (35)	458 (41)	331 (27)	
**Main occupation before age 60**						< 0.001
Agriculture	3194 (57)	1016 (71)	1022 (54)	415 (39)	741 (62)	
Professional/managerial	980 (18)	124 (9)	379 (20)	318 (30)	159 (13)	
Others	1396 (25)	288 (20)	479 (25)	335 (31)	294 (25)	
**Household income**						< 0.001
< 6000 Yuan	1254 (25)	422 (32)	349 (20)	148 (18)	335 (30)	
6000–19,999 Yuan	1033 (21)	306 (23)	345 (20)	153 (19)	229 (21)	
20,000–39,999 Yuan	1068 (21)	284 (21)	403 (24)	176 (21)	205 (19)	
≥ 40,000 Yuan	1626 (33)	327 (24)	617 (36)	347 (42)	335 (30)	
**Self-rated health**						< 0.001
Very bad	73 (1)	22 (1)	14 (1)	5 (0)	32 (2)	
Bad	748 (12)	196 (13)	153 (7)	87 (7)	312 (22)	
Average	2443 (38)	573 (38)	784 (34)	421 (34)	665 (46)	
Good	2396 (37)	569 (38)	957 (42)	525 (42)	345 (24)	
Very good	796 (12)	137 (9)	369 (16)	212 (17)	78 (5)	
**Cognitive impairment**						
MMSE score, mean (SD)	27.3 (3.7)	27.9 (3.1)	25.7 (4.9)	28.1 (2.7)	27.0 (3.8)	< 0.001
No	3471 (90)	897 (93)	831 (85)	1677 (96)	956 (91)	< 0.001
Yes	397 (10)	70 (7)	142 (15)	96 (4)	89 (9)	
**Disability**						< 0.001
No	5256 (79)	1097 (64)	2017 (90)	1015 (81)	1127 (79)	
Yes	1376 (21)	619 (36)	218 (10)	237 (19)	302 (21)	

* *P*-values were obtained from the Chi-square test. For comparison of MMSE score, *P*-value was obtained from the analysis of variance

**Table 2:** Sociodemographic characteristics of latent lifestyle classes for women (*N* = 8902)

Variables	Total (***N*** = 8902)	Health Promoting (***N*** = 2493)	Isolated and Health Harming (***N*** = 3027)	Restless (***N*** = 1513)	Restless and dismal (***N*** = 1869)	***P***-value
	N (%)	N (%)	N (%)	N (%)	N (%)	
**Age**						< 0.001
65–79	2654 (30)	385 (12)	1257 (52)	713 (45)	299 (18)	
≥ 80	6248 (70)	2806 (88)	1181 (48)	856 (55)	1405 (82)	
**Residence area**						< 0.001
Rural area	4056 (46)	1585 (50)	997 (41)	590 (38)	884 (52)	
Urban area	4846 (54)	1606 (50)	1441 (59)	979 (62)	820 (48)	
**Current marital status**						< 0.001
Not in marriage	6367 (30)	2797 (12)	1258 (52)	941 (45)	1371 (18)	
In marriage	2389 (70)	298 (88)	1162 (48)	614 (55)	315 (82)	
**Living status**						< 0.001
Alone	345 (5)	99 (4)	105 (5)	86 (7)	55 (4)	
With others	6830 (95)	2457 (96)	1936 (95)	1174 (93)	1263 (96)	
**Education**						< 0.001
Illiterate	5216 (68)	2244 (82)	1036 (48)	717 (52)	1219 (83)	
Literate or primary school	1691 (22)	372 (14)	703 (33)	404 (29)	212 (14)	
Junior high and above	820 (11)	104 (4)	420 (19)	257 (19)	39 (3)	
**Main occupation before 60**						< 0.001
Agriculture	5064 (67)	1970 (74)	1227 (58)	782 (57)	1085 (75)	
Professional/managerial	443 (6)	70 (3)	221 (10)	131 (10)	21 (1)	
Others	2092 (28)	636 (24)	666 (32)	451 (33)	339 (23)	
**Household income**						< 0.001
< 6000 Yuan	1789 (27)	639 (27)	357 (21)	287 (26)	506 (38)	
6000–19,999 Yuan	1322 (20)	519 (22)	319 (19)	178 (16)	306 (23)	
20,000–39,999 Yuan	1520 (23)	596 (25)	392 (23)	264 (24)	268 (20)	
≥ 40,000 Yuan	1913 (29)	632 (26)	649 (38)	363 (33)	269 (20)	
**Self-rated health**						< 0.001
Very bad	119 (2)	28 (1)	21 (1)	16 (1)	54 (4)	
Bad	1095 (14)	259 (10)	188 (8)	247 (16)	401 (27)	
Average	3145 (40)	904 (36)	829 (35)	732 (48)	680 (46)	
Good	2704 (34)	1009 (40)	976 (41)	431 (28)	288 (20)	
Very good	832 (11)	312 (12)	357 (15)	110 (7)	53 (4)	
**Cognitive impairment**						
MMSE score, mean (SD)	25.7 (4.7)	27.3 (3.5)	24.1 (5.4)	26.8 (3.8)	24.0 (5.2)	0.11
No	4899 (88)	1805 (93)	1223 (81)	1135 (92)	736 (83)	< 0.001
Yes	654 (12)	128 (7)	283 (19)	93 (8)	150 (17)	
**Disabled**						< 0.001
No	5774 (67)	1612 (53)	1950 (83)	1231 (81)	981 (59)	
Yes	2812 (33)	1453 (47)	404 (17)	284 (19)	671 (41)	

* *P*-values were obtained from the Chi-square test. For comparison of MMSE score, *P*-value was obtained from the analysis of variance

### Association between latent lifestyle classes and disability

The relationship between lifestyle classes and disability is shown in Table 3. In Model 1, women in the “Isolated and Health Harming” (OR = 1.77, 95% CI: 1.41–2.22) and “Restless and Dismal” (OR = 1.56, 95% CI: 1.21–2.02) classes showed higher disability risks compared with those in the “Health Promoting” class, after accounting for age, marital status, residence, living status, education, occupation before 60, household income, and self-rated health (Table 3). For men, compared with the “Health Promoting” class, the “Isolated and Health Harming” class (OR = 1.43, 95% CI: 1.05–1.95) showed higher risk of disability while the “Discordant” class had lower risk (OR = 0.53, 95% CI: 0.38–0.73) (Table 3).

**Table 3:** Multivariable logistic regression of the association between latent lifestyle classes and disability, stratified by sex

Variables	Model 1		Model 2	
	Men	Women	Men	Women
**Lifestyle class for men**				
Health Promoting	ref		ref	
Isolated and Health Harming	1.43* (1.05, 1.95)		1.34 (0.98, 1.83)	
Discordant	0.53** (0.38, 0.73)		0.52** (0.37, 0.72)	
Restless and Dismal	0.91 (0.65, 1.27)		0.88 (0.63, 1.23)	
**Lifestyle class for women**				
Health Promoting		ref		ref
Isolated and Health Harming		1.77** (1.41, 2.22)		1.88** (1.46, 2.43)
Restless		0.91 (0.69, 1.21)		1.11 (0.84, 1.47)
Restless and Dismal		1.56** (1.21, 2.02)		1.67** (1.27, 2.20)
**Age**				
65–79	ref	ref	ref	ref
≥ 80	4.24** (3.18, 5.72)	6.36** (4.81, 8.49)	3.99** (2.97, 5.35)	5.97** (4.49, 7.94)
**Residence area**				
Rural area	ref	ref		
Urban area	1.26* (1.01, 1.57)	1.29** (1.09, 1.53)	1.25 (1.00, 1.56)	1.29** (1.09, 1.53)
**Current marital status**				
Not in marriage	ref	ref	ref	ref
In marriage	0.41** (0.33, 0.52)	0.39** (0.31, 0.50)	0.43** (0.34, 0.54)	0.41** (0.32, 0.52)
**Living status**				
Alone	ref	ref	ref	ref
With others	0.69 (0.42, 1.14)	0.58** (0.38, 0.86)	0.69 (0.42, 1.14)	0.58** (0.39, 0.87)
**Education**				
Illiterate	ref	ref	ref	ref
Literate or primary school	0.65** (0.51, 0.82)	0.66** (0.52, 0.83)	0.68** (0.53, 0.86)	0.68** (0.54, 0.86)
Junior high and above	0.57** (0.40, 0.80)	0.61* (0.38, 0.96)	0.50** (0.35, 0.71)	0.54* (0.34, 0.87)
**Main occupation before 60**				
Agriculture	ref	ref	ref	ref
Professional/managerial	2.48** (1.72, 3.58)	1.68 (0.98, 2.86)	2.54** (1.75, 3.68)	1.72* (1.00, 2.94)
Others	1.77** (1.36, 2.30)	1.60** (1.32, 1.94)	1.72** (1.32, 2.25)	1.61** (1.32, 1.95)
**Household income**				
< 6000 Yuan	ref	ref	ref	ref
6000–19,999 Yuan	1.15 (0.82, 1.60)	1.31* (1.02, 1.69)	1.16 (0.83, 1.63)	1.36* (1.06, 1.76)
20,000–39,999 Yuan	0.97 (0.70, 1.35)	1.07 (0.85, 1.36)	0.98 (0.70, 1.37)	1.10 (0.86, 1.40)
≥ 40,000 Yuan	1.04 (0.76, 1.42)	1.10 (0.87, 1.38)	1.07 (0.78, 1.46)	1.14 (0.90, 1.44)
**Self-rated health**				
Very bad	ref	ref	ref	ref
Bad	0.36** (0.16, 0.78)	0.59 (0.30, 1.12)	0.34** (0.16, 0.74)	0.58 (0.30, 1.13)
Average	0.17** (0.08, 0.37)	0.26** (0.13, 0.49)	0.18** (0.08, 0.38)	0.26** (0.14, 0.49)
Good	0.12** (0.05, 0.25)	0.21** (0.11, 0.40)	0.12** (0.06, 0.26)	0.21** (0.11, 0.40)
Very good	0.12** (0.05, 0.28)	0.22** (0.11, 0.43)	0.13** (0.06, 0.29)	0.22** (0.11, 0.44)
**Cognitive impairment**				
No	–	–	ref	ref
Yes	–	–	2.45** (1.72, 3.50)	2.14** (1.67, 2.74)

Values were odds ratios with 95% confidence interval in brackets

*P < 0.05; **P < 0.01; ***P < 0.001

In Model 2 that additionally adjusted for cognitive impairment status, the magnitude of the associations between lifestyle classes and disability was lessened (Table 3). Nevertheless, the risk of disability remained statistically higher in women who were in the “Isolated and Health Harming” class (OR = 1.88, 95% CI: 1.46–2.43) and “Restless and Dismal” class (OR = 1.67, 95% CI: 1.27–2.20) relative to the “Health Promoting” class. The risk of disability was lower in men who were in the “Discordant” class compared to the “Health Promoting” class (OR = 0.52, 95% CI: 0.37–0.72), after additionally adjusting for cognitive impairment (Table 3). Also, younger participants tended to have higher education (Supplemental Tables 6, Additional File 1). In sensitivity analyses stratified by age, the associations between lifestyle classes and disability were numerically stable across age groups, with the strongest associations in the 75–84 years group for women and the 65–74 years group for men (Supplemental Tables 7, Additional File 1).

## Discussion

In this analysis, we identified four latent classes of lifestyle for older women and men based on 16 lifestyle factors in a nationally representative sample of older adults in China. We included a broad range of lifestyle factors that have been associated with disability [1]. By including factors in the behavioral, mental and social domains of lifestyle, our analysis is capable of distinguishing among people who maintain a healthy lifestyle in all aspects and those who simply perform well in one or two domains. In addition, we advance previous research on lifestyle patterns by showing that the lifestyle patterns were significantly associated with the risk of disability among older Chinese adults. The findings can deepen our understanding of the common lifestyle patterns in older adults, and provide important evidence for developing interventions to prevent disability.

In our study, the four latent classes of lifestyle patterns for women were “Health Promoting”, “Isolated and Health Harming”, “Restless” and “Restless and Dismal”; the latent classes for men were “Health Promoting”, “Isolated and Health Harming”, “Discordant” and “Restless and Dismal”. Our results are consistent with previous studies that have also shown that different patterns exist in lifestyle, health beliefs, and behaviors for men and women [13]. The different latent classes of lifestyles in men and women may be strongly influenced by social contexts that moderate people’s perception and maintenance of health behaviors, especially diet and physical activity [37, 38]. Education and prevention programs for lifestyle need to consider sex differences in lifestyles.

For instance, among people who tended to follow health-harming behaviors, men were more likely to show good social engagement (“Discordant” class) compared to women (“Isolated and Health Harming” class). Our finding is consistent with a study in Taiwan, which suggests that social engagement is positively correlated with alcohol consumption in men [39]. In addition, women are more likely to have mental health problems compared with men [40]. Previous studies have shown that substance use, such as smoking and alcohol drinking, increases when people are lonely or depressed [41]. Also, women tended to be more concerned about health issues than men [42].

As 81% of the current sample was illiterate or only attended primary school, the influence of education on lifestyle choices needs to be considered when extrapolating our findings to other populations. While it is generally believed that people with higher education are more likely to follow healthier lifestyles, their relationship is not necessarily causative. For instance, a study in the Philippines (*n* = 1064) showed that it is not the years of education, but health knowledge, that promotes a healthy lifestyle [43]. Moreover, a British study (*n* = 9003) showed that the improvement in education following education reform did not significantly improve health knowledge [44]. In our analyses, despite the stark differences in education across age groups, the observed association between lifestyle and disability was generally comparable in younger and older participants. Our findings suggest that lifestyle factors may exert an independent effect on physical functioning of older adults, irrespective of education level. Therefore, it is important to promote health knowledge in older adults of all education levels to reduce or delay disability.

For women, a lifestyle characterized by social isolation was strongly associated with disability. Although previous studies have found that social relations and social support are associated with disability in older adults, it is not clear whether social support directly leads to disability [21, 45]. Our study found that older adults with poor social relations tend to have health-harming behaviors, such as smoking, alcohol drinking, and non-exercise, which may increase the risk of disability. Thus, appropriate health interventions can be tailored to socially isolated groups, such as advocating abstinence and exercise, to reduce the risk of disability and improve the quality of life for older adults.

For men, the class of “Discordant” had a lower risk of disability compared to the class of “Health Promoting”. A plausible explanation is that health-promoting behaviors such as exercise and good social engagement may carry positive effects that offset the negative effects of smoking and drinking. In addition, compared with the class of “Health Promoting”, the class of “Discordant” was younger, which may lead to a lower risk of disability in the class of “Discordant”. However, in multivariable analyses adjusting for age, the “Discordant” class still showed lower risk of disability compared to the “Health Promoting” class. Another possible explanation is that the disabled older adults may be motivated to change unfavorable lifestyles to delay the process of disability, leading to a higher risk of disability in the class of “Health Promoting” than in the class of “Discordant”.

Compared with men, women with poorer mental health and sleep quality may be at higher risk of disability. Previous studies have shown that a normal mental state is a protective factor for disability in older adults, and depression may aggravate the degree of disability [46]. Depression tended to associate with chronic diseases such as diabetes and hypertension, which may increase the risk of disability [47, 48]. Further, depression aggravates functional disability in patients with chronic diseases and affects the ability of daily instrumental activities in varying degrees [49]. Therefore, it is necessary to take social and psychological intervention measures, such as providing opportunities based on the abilities and preferences of older adults to participate in meaningful social activities, to reduce the psychological problems of older adults.

To the best of our knowledge, this study is one of the first to analyze the relationship between multidimensional lifestyle patterns and disability. However, our study suffers from several limitations. First, as this study is a cross-sectional study, the causal relationship between lifestyle and disability is not clear. Second, we cannot assess the impact of lifestyle changes on disability. For example, since smoking, alcohol drinking and exercise data were assessments of recent conditions, the effects of quitting smoking and alcohol cessation on disability cannot be evaluated. Third, the observed association could be due to reverse causation, that is, the disabled older adults may be more motivated to change their lifestyle, leading to an association between a healthier lifestyle and higher risk of disability. Since the effects of lifestyle are likely cumulative, more years of data should be collected to assess the impact of lifestyle and its change on disability. Fourth, cognitive impairment may confound the observed association as it may be associated with both lifestyle and disability, and may introduce measurement errors in lifestyle factors. In analyses adjusting for cognitive impairment, lifestyle classes were still associated with disability albeit reduced magnitude of association. However, it is possible that unmeasured confounding may have contributed to the observed association. Fifth, since the majority of the sample was illiterate or only attended primary education, our findings may not be generalizable to other populations with higher education levels. Lastly, the impact of measurement errors cannot be completely ignored, even if the survey was conducted face-to-face by trained personnel. Furthermore, using binary values to indicate alcohol consumption and exercise poses a significant limitation as a person who walks 15 min once per week could tick “yes” for exercise, which would obviously differ greatly from someone else who did a combination of weight-bearing and aerobic exercise multiple times per week. Future research needs more objective lifestyle measurements to identify lifestyle classes and assess the association between lifestyle and disability.

## Conclusions

In conclusion, we identified different lifestyle patterns in men and women over 65 years old in China. An unhealthy lifestyle characterized by health-harming behaviors and poor mental and social functioning was associated with a higher risk of disability. For women, the class of “Isolated and Health Harming” and the class of “Restless and Dismal” had higher risk of disability, while the class of “Discordant” had lower risk of disability in men. These results suggest that education and interventions for disability prevention should not only focus on health behaviors but also promote mental health and social engagement. In addition, sex differences in lifestyle pattern need to be considered when formulating interventions to prevent disability.

## Supplementary Information

**Additional file 1: Supplemental Table 1.** Categorization of lifestyle variables for latent class analysis. **Supplemental Table 2.** Sample characteristics of the analytical sample (*N*= 15,771). **Supplemental Table 3.** Model fitting statistics for group number selection. **Supplemental Table 4.** Test of measurement invariance for lifestyle latent classes between women and men. **Supplemental Table 5.** Sociodemographic characteristics by sex and disability status (*N*= 15,771). **Supplemental Table 6.** Distribution of education levels by age groups. **Supplemental Table 7.** Multivariable logistic regression of the association between latent lifestyle classes and disability, stratified by age groups.

## Data Availability

The analytical dataset used in this study is a publicly available dataset released by the CLHLS. Information about the data source and available data are found at https://www.icpsr.umich.edu/icpsrweb/DSDR/studies/36179. Researchers can obtain these data after submitting a data use agreement to the CLHLS team.

## Abbreviations

CLHLS: Chinese Longitudinal Healthy Longevity Survey
WHO: World Health Organization
ICF: International Classification of Functioning, Disability, and Health
CES-D: Center for Epidemiologic Studies Depression Scale
GAD: Generalized anxiety disorder
MMSE: Mini-mental state examination
ADL: Activities of daily living
LCA: Latent class analysis
EM: Expectation-maximization algorithm
BIC: Bayesian information criterion
aBIC: Adjusted BIC
AIC: Akaike’s information criterion
cAIC: Consistent Akaike’s information criterion
