# Supplementary material for: Sex differences in the association between latent class of lifestyle and disability among older adults in China
Source: BMC Geriatr. 2021 Mar 18;21:188. doi: 10.1186/s12877-021-02087-z (PMC7976722; doi:10.1186/s12877-021-02087-z)
Supplement: Supplementary file 1 — Additional file 1: Supplemental Table 1. Categorization of lifestyle variables for latent class analysis. Supplemental Table 2. Sample characteristics of the analytical sample (N= 15,771). Supplemental Table 3. Model fitting statistics for group number selection. Supplemental Table 4. Test of measurement invariance for lifestyle latent classes between women and men. Supplemental Table 5. Sociodemographic characteristics by sex and disability status (N= 15,771). Supplemental Table 6. Distribution of education levels by age groups. Supplemental Table 7. Multivariable logistic regression of the association between latent lifestyle classes and disability, stratified by age groups. [file 12877_2021_2087_MOESM1_ESM.docx]

**Sex differences in the association between latent class of lifestyle and disability among older adults in China**

Zaixing Shi, PhD^1,2†^, Jianlin Lin, MM^2†^, Jian Xiao, MM^2^ and Ya Fang, MD, PhD^1,2^

1. State Key Laboratory of Molecular Vaccinology and Molecular Diagnostics, School of Public Health, Xiamen University, Xiamen, China;

2. Key Laboratory of Health Technology Assessment of Fujian Province, School of Public Health, Xiamen University, Xiamen, China

† These authors contributed equally to this work

**Corresponding Author**:

Ya Fang, MD, PhD

Professor

School of Public Health, Xiamen University

Xiang’an South Road, Xiamen 361102, China

Email: fangya@xmu.edu.cn, Tel.: +86-592-2880636

**Supplemental Files**

**Supplemental Table 1. Categorization of lifestyle variables for latent class analysis.**

| **Lifestyle dimension** | **Variable** | **Value assignment** |
| --- | --- | --- |
| **Health behaviors** | Sleep quality | 1= bad  2= moderate  3= good |
|  | Sleep duration | 1= <7 hours/day  2= 7-8 hours/day  3= >8 hours/day |
|  | Consumption of fruits | 1= insufficient (every day or almost every day or quite often)  2= sufficient (occasionally or rarely or never) |
|  | Consumption of vegetables |  |
|  | Smoking | 1= yes  2= no |
|  | Alcohol drinking |  |
|  | Exercise |  |
|  | Physical examination |  |
|  | Frequency of brushing teeth | 1= less than twice per day  2= twice or more per day |
|  | Hypertension management | 1= not diagnosed  2= diagnosed but not treated  3= diagnosed and treated |
|  | Diabetes management |  |
|  | Dyslipidemia management |  |
| **Psychological wellbeing** | Depression | 1= no depression (score < 27)  2= depression (score ≥27) |
|  | Anxiety | 1= no anxiety (score < 14)  2= anxiety (score ≥14) |
| **Social engagement** | Structural relationship | 1= poor structural relationships (score ≤ 2)  2= good structural relationships (score > 2) |
|  | Functional relationship | 1= poor functional relationships (score ≤ 10)  2= good functional relationships (score > 10) |

**Supplemental Table 2 Sample characteristics of the analytical sample (N= 15,771)**

| **Variables** | **N（%）** |
| --- | --- |
| **Age** |  |
| 65-79 | 5352 (34) |
| ≥80 | 10419 (66) |
| **Sex** |  |
| Men | 6869 (44) |
| Women | 8902 (56) |
| **Residence area** |  |
| Rural area | 7034 (45) |
| Urban area | 8737 (55) |
| **Current marital status** |  |
| Not in marriage | 9180 (34) |
| In marriage | 6337 (66) |
| **Living status** |  |
| Alone | 572 (4) |
| With others | 12415 (96) |
| **Education** |  |
| Illiterate | 6790 (50) |
| Literate or primary school | 4195 (31) |
| Junior high and above | 2501 (19) |
| **Main occupation before 60** |  |
| Agriculture | 8258 (63) |
| Professional/managerial | 1423 (11) |
| Others | 3488 (26) |
| **Household income** |  |
| Lower than 6,000 Yuan | 3043 (26) |
| 6,000 Yuan - | 2355 (21) |
| 20,000 Yuan - | 2588 (22) |
| Higher than 40,000 Yuan | 3539 (31) |
| **self-rated health** |  |
| Very bad | 192 (1) |
| Bad | 1843 (13) |
| Average | 5588 (39) |
| Good | 5100 (36) |
| Very good | 1628 (11) |
| **Cognitive impairment** |  |
| No | 8370 (89) |
| Yes | 1051 (11) |
| **Disabled** |  |
| No | 11030 (72) |
| Yes | 4188 (28) |

**Supplemental Table 3 Model fitting statistics for group number selection**

| **Sex** | **Indicator** | **2 classes** | **3 classes** | **4 classes** | **5 classes** | **6 classes** | **7 classes** | **8 classes** | **9 classes** | **10 classes** |
| --- | --- | --- | --- | --- | --- | --- | --- | --- | --- | --- |
| **Men** | ABIC | 7034.56 | 6312.21 | 5897.95 | 5753.21 | 5599.39 | 5535.36 | 5534.98 | 5550.69 | 5584.67 |
|  | AIC | 6915.78 | 6132.35 | 5657 | 5451.17 | 5236.27 | 5111.16 | 5049.69 | 5004.32 | 4977.21 |
|  | BIC | 7145.78 | 6480.63 | 6123.56 | 6036.02 | 5939.4 | 5932.57 | 5989.38 | 6062.3 | 6153.47 |
|  | CAIC | 7180.78 | 6533.63 | 6194.56 | 6125.02 | 6046.4 | 6057.57 | 6132.38 | 6223.3 | 6332.47 |
| **Women** | ABIC | 6261.91 | 5412.32 | 5134.89 | 4945.83 | 4885.03 | 4826.52 | 4831.16 | 4846.83 | 4865.86 |
|  | AIC | 6138.79 | 5225.88 | 4885.12 | 4632.75 | 4508.63 | 4386.8 | 4328.12 | 4280.47 | 4236.18 |
|  | BIC | 6373.13 | 5580.74 | 5360.51 | 5228.65 | 5225.05 | 5223.73 | 5285.57 | 5358.44 | 5434.67 |
|  | CAIC | 6408.13 | 5633.74 | 5431.51 | 5317.65 | 5332.05 | 5348.73 | 5428.57 | 5519.44 | 5613.67 |

**Supplemental Table 4 Test of measurement invariance for lifestyle latent classes between women and men**

|  | **Log likelihood** | **DF** | ***G^2^*** | **AIC** | **BIC** |
| --- | --- | --- | --- | --- | --- |
| **Model 1: Item-response probabilities free to vary across sex** | -98233.46 | 995153.00 | 26355.91 | 26703.91 | 27979.07 |
| **Model 2：Item-response probabilities constrained equal across sex** | -99057.03 | 995237.00 | 28003.05 | 28183.05 | 28842.62 |
| $G_{2}^{2}-G_{1}^{2}=1647.14,df=84,P<0.001$ | | | | | |

* P-values were obtained from Chi-square test.

**Supplemental Table 5 Sociodemographic characteristics by sex and disability status. (N= 15,771)**

|  | **Men** | | | **Women** | | |
| --- | --- | --- | --- | --- | --- | --- |
|  | **Nondisabled** | **Ddisabled** | **P-value** | **Nondisabled** | **Disabled** | **P-value*** |
|  | **N（%）** | **N（%）** |  | **N（%）** | **N（%）** |  |
| **Age** |  |  | <0.001 |  |  | <0.001 |
| 65-79 | 2452 (47) | 130 (9) |  | 2370 (41) | 146 (5) |  |
| ≥ 80 | 2804 (53) | 1246 (91) |  | 3404 (59) | 2666 (95) |  |
| **Residence area** |  |  | <0.001 |  |  | <0.001 |
| Rural area | 2366 (45) | 497 (36) |  | 2756 (48) | 1175 (42) |  |
| Urban area | 2890 (55) | 879 (64) |  | 3018 (52) | 1637 (58) |  |
| **Current marital status** |  |  | <0.001 |  |  | <0.001 |
| Not in marriage | 1830 (47) | 915 (9) |  | 3680 (41) | 2523 (5) |  |
| In marriage | 3372 (53) | 442 (91) |  | 2042 (59) | 258 (95) |  |
| **Living status** |  |  | <0.001 |  |  | <0.001 |
| Alone | 110 (3) | 111 (9) |  | 131 (3) | 205 (8) |  |
| With others | 4289 (97) | 1135 (91) |  | 4311 (97) | 2330 (92) |  |
| **Education** |  |  | <0.001 |  |  | <0.001 |
| Illiterate | 1040 (24) | 494 (40) |  | 3024 (61) | 2048 (82) |  |
| Literate or primary school | 1968 (45) | 460 (37) |  | 1332 (27) | 308 (12) |  |
| Junior high and above | 1336 (31) | 282 (23) |  | 626 (13) | 154 (6) |  |
| **Main occupation before 60** |  |  | <0.001 |  |  | <0.001 |
| Agriculture | 2492 (59) | 608 (51) |  | 3349 (68) | 1566 (63) |  |
| Professional/managerial | 699 (17) | 249 (21) |  | 312 (6) | 113 (5) |  |
| Others | 1010 (24) | 337 (28) |  | 1231 (25) | 797 (32) |  |
| **Household income** |  |  | 0.017 |  |  | <0.001 |
| Lower than 6,000 Yuan | 990 (26) | 225 (23) |  | 1241 (29) | 494 (24) |  |
| 6,000 Yuan - | 814 (21) | 191 (20) |  | 831 (19) | 462 (22) |  |
| 20,000 Yuan - | 843 (22) | 197 (20) |  | 997 (23) | 494 (24) |  |
| Higher than 40,000 Yuan | 1223 (32) | 359 (37) |  | 1209 (28) | 641 (31) |  |
| **Self-rated health** |  |  | <0.001 |  |  | <0.001 |
| Very bad | 34 (1) | 36 (3) |  | 51 (1) | 65 (3) |  |
| Bad | 491 (10) | 235 (21) |  | 663 (12) | 408 (20) |  |
| Average | 1928 (37) | 437 (40) |  | 2264 (41) | 773 (37) |  |
| Good | 2026 (39) | 297 (27) |  | 1991 (36) | 631 (31) |  |
| Very good | 669 (13) | 101 (9) |  | 614 (11) | 186 (9) |  |
| **Cognitive impairment** |  |  | <0.001 |  |  | <0.001 |
| No | 3679 (87) | 223 (58) |  | 3988 (84) | 300 (47) |  |
| Yes | 532 (13) | 164 (42) |  | 737 (16) | 338 (53) |  |

* P-values were obtained from Chi-square test.

**Supplemental Table 6 Distribution of education levels by age groups**

* P-values were obtained from the Chi-square test.

|  | **Men** | | | | **Women** | | | |
| --- | --- | --- | --- | --- | --- | --- | --- | --- |
| **Age group** | **Illiterate**  **N(%)** | **Literate or primary school**  **N(%)** | **Junior high and above**  **N(%)** | **P-value** | **Illiterate**  **N(%)** | **Literate or primary school**  **N(%)** | **Junior high and above**  **N(%)** | **P-value*** |
| **65-74 years** | 156 (10) | 673 (45) | 671 (45) | <0.001 | 500 (33) | 621 (42) | 374 (25) | <0.001 |
| **75-84 years** | 378 (24) | 715 (45) | 494 (31) |  | 1027 (57) | 531 (29) | 244 (14) |  |
| **85+ years** | 1040 (39) | 1116 (42) | 516 (19) |  | 3689 (83) | 539 (12) | 202 (5) |  |

**Supplemental Table 7 Multivariable logistic regression of the association between latent lifestyle classes and disability, stratified by age groups**

| **Variables** | **Men** | | | **Women** | | |
| --- | --- | --- | --- | --- | --- | --- |
|  | **65-74 years** | **75-84 years** | **85+ years** | **65-74 years** | **75-84 years** | **85+ years** |
| **Lifestyle class for men** |  |  |  |  |  |  |
| Health Promoting | ref | ref | ref |  |  |  |
| Isolated and Health Harming | 2.41 (0.71, 8.23) | 1.48 (0.65, 3.38) | 1.17 (0.80, 1.69) |  |  |  |
| Discordant | 0.63 (0.21, 1.92) | 0.51 (0.24, 1.11) | 0.52** (0.35, 0.78) |  |  |  |
| Restless and Dismal | 1.36 (0.44, 4.20) | 1.32 (0.63, 2.77) | 0.79 (0.52, 1.21) |  |  |  |
| **Lifestyle class for women** | |  |  |  |  |  |
| Health Promoting |  |  |  | ref | ref | ref |
| Isolated and Health Harming | |  |  | 2.09 (0.45, 9.63) | 2.36* (1.16, 4.77) | 1.35 (1.00, 1.84) |
| Restless |  |  |  | 1.99 (0.71, 5.53) | 1.71 (0.90, 3.26) | 0.85 (0.60, 1.22) |
| Restless and Dismal |  |  |  | 2.05 (0.65, 6.51) | 2.83** (1.43, 5.59) | 1.24 (0.89, 1.73) |
| **Residence area** |  |  |  |  |  |  |
| Rural area | ref | ref | ref | ref | ref | ref |
| Urban area | 1.13 (0.53, 2.40) | 1.64 (0.97, 2.79) | 1.08 (0.83, 1.42) | 1.01 (0.45, 2.27) | 1.50 (0.95, 2.37) | 1.26* (1.04, 1.53) |
| **Current marital status** |  |  |  |  |  |  |
| Not in marriage | ref | ref | ref | ref | ref | ref |
| In marriage | 10.05 (0.67, 150.38) | 0.93 (0.49, 1.75) | 0.43** (0.32, 0.56) | 1.52 (0.50, 4.63) | 1.20 (0.76, 1.88) | 0.30** (0.21, 0.44) |
| **Living status** |  |  |  |  |  |  |
| Alone | ref | ref | ref | ref | ref | ref |
| With others | 0.01** (0.00, 0.07) | 1.14 (0.22, 5.95) | 0.85 (0.49, 1.48) | 0.09** (0.02, 0.48) | 0.25** (0.10, 0.64) | 0.80 (0.50, 1.29) |
| **Education** |  |  |  |  |  |  |
| Illiterate | ref | ref | ref | ref | ref | ref |
| Literate or primary school | 1.00 (0.36, 2.79) | 0.64 (0.35, 1.18) | 0.67** (0.51, 0.89) | 0.34* (0.14, 0.82) | 1.05 (0.65, 1.69) | 0.79 (0.58, 1.07) |
| Junior high and above | 0.60 (0.18, 2.01) | 0.92 (0.45, 1.90) | 0.47** (0.30, 0.74) | 0.65 (0.21, 2.02) | 0.27* (0.09, 0.80) | 0.93 (0.46, 1.91) |
| **Main occupation before 60** | |  |  |  |  |  |
| Agriculture | ref | ref | ref | ref | ref | ref |
| Professional/managerial | 2.19 (0.59, 8.09) | 1.11 (0.46, 2.68) | 3.20** (2.03, 5.05) | 0.26 (0.02, 4.52) | 2.07 (0.66, 6.45) | 1.47 (0.72, 3.00) |
| Others | 0.97 (0.36, 2.57) | 1.25 (0.66, 2.35) | 1.91** (1.38, 2.64) | 1.00 (0.39, 2.59) | 1.15 (0.68, 1.95) | 1.76** (1.40, 2.21) |
| **Household income** |  |  |  |  |  |  |
| <6,000 yuan | ref | ref | ref | ref | ref | ref |
| 6,000-19,999 yuan | 0.98 (0.38, 2.54) | 0.76 (0.32, 1.83) | 1.14 (0.75, 1.73) | 0.32 (0.08, 1.22) | 1.24 (0.64, 2.41) | 1.45* (1.08, 1.95) |
| 20,000-39,999 yuan | 0.38 (0.11, 1.24) | 1.50 (0.72, 3.14) | 0.93 (0.62, 1.40) | 0.58 (0.20, 1.70) | 1.43 (0.78, 2.60) | 1.05 (0.79, 1.38) |
| ≥40,000 yuan | 0.75 (0.26, 2.17) | 1.23 (0.60, 2.50) | 1.07 (0.73, 1.57) | 0.97 (0.37, 2.58) | 1.23 (0.68, 2.22) | 1.12 (0.85, 1.47) |
| **Self-rated health** |  |  |  |  |  |  |
| Very bad | ref | ref | ref | ref | ref | ref |
| Bad | 0.39 (0.08, 1.87) | 0.93 (0.17, 5.00) | 0.17* (0.04, 0.68) | 0.14 (0.01, 1.32) | 0.95 (0.23, 4.00) | 0.56 (0.24, 1.32) |
| Average | 0.13** (0.03, 0.61) | 0.31 (0.06, 1.64) | 0.12** (0.03, 0.47) | 0.04** (0.00, 0.38) | 0.46 (0.11, 1.88) | 0.27** (0.12, 0.62) |
| Good | 0.05** (0.01, 0.28) | 0.12* (0.02, 0.70) | 0.09** (0.02, 0.35) | 0.01** (0.00, 0.15) | 0.26 (0.06, 1.12) | 0.22** (0.10, 0.50) |
| Very good | 0.07** (0.01, 0.47) | 0.34 (0.06, 2.07) | 0.09** (0.02, 0.36) | 0.01** (0.00, 0.24) | 0.22 (0.04, 1.12) | 0.24** (0.10, 0.57) |
| **Cognitive impairment** |  |  |  |  |  |  |
| No | ref | ref | ref | ref | ref | ref |
| Yes | 4.55* (1.07, 19.25) | 2.17 (0.96, 4.92) | 2.14** (1.43, 3.19) | 0.00 (0.00,  Inf) | 4.44** (2.10, 9.39) | 1.91** (1.47, 2.48) |

Values were odds ratios with 95% confidence interval in brackets.

*P < 0.05; **P < 0.01; ***P < 0.001.
